# BAYSIC: a Bayesian method for combining sets of genome variants with improved specificity and sensitivity

**DOI:** 10.1186/1471-2105-15-104

**Published:** 2014-04-12

**Authors:** Brandi L Cantarel, Daniel Weaver, Nathan McNeill, Jianhua Zhang, Aaron J Mackey, Justin Reese

**Affiliations:** 1 Baylor Health, Baylor Institute for Immunology Research, Dallas, TX 75204, USA; 2 Genformatic, LLC, Austin, TX 78731, USA; 3 Institute for Applied Cancer Science, University of Texas MD Anderson Cancer Center, Houston, TX 77030, USA; 4 Center for Public Health Genomics, University of Virginia School of Medicine, Charlottesville, VA 22908, USA

**Keywords:** SNP, Genome variants, Bayesian, Latent class analysis, Cancer, Somatic mutation

## Abstract

**Background:**

Accurate genomic variant detection is an essential step in gleaning medically useful information from genome data. However, low concordance among variant-calling methods reduces confidence in the clinical validity of whole genome and exome sequence data, and confounds downstream analysis for applications in genome medicine.

Here we describe BAYSIC (BAYeSian Integrated Caller), which combines SNP variant calls produced by different methods (*e.g.* GATK, FreeBayes, Atlas, SamTools, *etc.*) into a more accurate set of variant calls. BAYSIC differs from majority voting, consensus or other *ad hoc* intersection-based schemes for combining sets of genome variant calls. Unlike other classification methods, the underlying BAYSIC model does not require training using a “gold standard” of true positives. Rather, with each new dataset, BAYSIC performs an unsupervised, fully Bayesian latent class analysis to estimate false positive and false negative error rates for each input method. The user specifies a posterior probability threshold according to the user’s tolerance for false positive and false negative errors; lowering the posterior probability threshold allows the user to trade specificity for sensitivity while raising the threshold increases specificity in exchange for sensitivity.

**Results:**

We assessed the performance of BAYSIC in comparison to other variant detection methods using ten low coverage (~5X) samples from The 1000 Genomes Project, a tumor/normal exome pair (40X), and exome sequences (40X) from positive control samples previously identified to contain clinically relevant SNPs. We demonstrated BAYSIC’s superior variant-calling accuracy, both for somatic mutation detection and germline variant detection.

**Conclusions:**

BAYSIC provides a method for combining sets of SNP variant calls produced by different variant calling programs. The integrated set of SNP variant calls produced by BAYSIC improves the sensitivity and specificity of the variant calls used as input. In addition to combining sets of germline variants, BAYSIC can also be used to combine sets of somatic mutations detected in the context of tumor/normal sequencing experiments.

## Background

The decreasing cost of producing sequence data has made the sequencing of genomes routine. Researchers use genome resequencing to identify how genomic changes are related to phenotype in their organism of interest. In the case of humans and certain other genomes (e.g., dogs, cats and livestock), resequencing projects aim to associate genetic changes to disease risk, medical treatment efficacy or other traits of interest. In some applications it is desirable to detect *de novo* somatic mutations, which may affect disease progression, prognosis and therapy. In other applications like genomic medicine for cancer, genomic variants in normal tissue can be compared to genomic variants of the tumor to identify relevant somatic mutations.

However, the accurate detection of single nucleotide variants (SNPs) and small insertions or deletions (indels) is not trivial. There is no standard protocol for detecting SNP predictions with the highest sensitivity and specificity. Each algorithm used in SNP detection creates a different balance of sensitivity and specificity, to either increase the number of true positives at the cost of additional false positives or decrease the number of false positives at the cost of reducing the number of true positives. Additionally, many variant calling algorithms do not provide quantitative values for filtering the VCF output file, or if they do provide users with numerical values for quality score filtering, it often remains unclear to the naïve user what is an appropriate filter. Variant calling programs like GATK and Atlas provide only qualitative values for filtering, with language like “PASS” or “LowQual”. In addition, some algorithms, e.g. GATK, recommend the user include many samples in order to recalibrate quality scores or classify SNPs with distinctions between PASS and LowQual, and thereby increase variant call accuracy.

Maximal sensitivity is desirable to minimize false negative calls and therefore avoid missing true mutations. The consequences of not detecting real variation include: 1) failure to diagnosis or detect real disease and correctly predict elevated or reduced risk for medical problems or potential drug effects, and 2) excess mortality or suffering because of nonintervention or non-optimal treatment. Maximal specificity is also essential to minimize false positive calls and thereby avoid erroneous over-diagnosis and the time, patient distress and expense of confirmatory testing and potential morbidity from unneeded overtreatment. Unfortunately, any classifier performing a nontrivial detection operation on real-world data achieves improved sensitivity only by accepting some elevated rate of false positives, and thus reduced specificity. This detection error tradeoff (DET) is an essential feature of detection task performance [1]. Because any single classifier has an inherent sensitivity versus specificity tradeoff, we hypothesized that more sophisticated methods for systematically integrating the output from multiple independent classifiers (here alternative methods of variant calling) – some with higher inherent sensitivity, some with higher intrinsic specificity - would result in overall improvement in the receiver operating characteristics of the BAYSIC integrated call set compared to the input call sets.

Managing sensitivity and specificity of variant calls is critical in projects using genomic data for clinical care [2]. Variant call accuracy may be affected by multiple factors, including systematic sequencing error, sequence read depth, allele variant fraction and position-specific error rate, among others. While there have been recent descriptions of other methods to improve variant call accuracy, including means of combining read mapping and variant call algorithms, these methods typically require training on a gold standard dataset considered to be the truth [3,4]. By contrast, BAYSIC is a completely unsupervised machine learning method. BAYSIC does not depend upon training and discordant call arbitration with validated data, yet still achieves gains in sensitivity and specificity over input call sets. Moreover, clinical genome sequencing often involves small sample numbers and/or variant calls in genomic regions with low sequencing coverage. For example, many clinical applications involve only trios of exomes or genomes, comparing SNPs differential between two healthy parents and a sick child for diagnosis and treatment selection. In other clinical cases, real SNPs could be missed in low read depth regions where the number of reads containing a SNP do not meet a strict *a priori* evidence threshold for inclusion in lists of putative clinically relevant variants [5].

Here we describe BAYSIC (**BAYeS**ian **I**ntegrated **C**aller), a novel algorithm that uses a Bayesian statistical method based on latent class analysis to combine variant sets produced by different bioinformatic packages (e.g., GATK, FreeBayes, Samtools) into a high-confidence set of genome variants. The strengths of BAYSIC are several. First, BAYSIC integrates data produced from multiple SNP callers, each with differing biases, and produces a call set with a posterior probability that is intuitive and can be used for quantitative filtering. Equally important, BAYSIC is a completely unsupervised method of clustering or classification and requires no training on a “gold standard” or validated data sets.

Third, BAYSIC performance improves along with the sensitivity or specificity gains of the input call sets. If new calling methods yield improved specificity and sensitivity, then BAYSIC will reap those rewards too. For example, in applications in which sensitivity is a priority, the BAYSIC posterior probability cutoff can be set low to minimize false negatives, and for applications in which specificity is a priority it can be set high to minimize false positives. BAYSIC run with a posterior probability threshold of 0.9 produces more sensitive and specific SNP prediction than any individual caller used as input.

## Implementation

### BAYSIC algorithm

The user provides variant calls from one or more variant calling programs of their choice in VCF format and, optionally, a posterior probability cutoff (default cutoff = 0.8). While not required, the user may also provide a VCF file containing the contents of third party database (e.g. dbSNP for germline variants or COSMIC for somatic mutations) as an additional source of variant information for BAYSIC.

False positive and false negative error rates for each evidence source (variant calling program (either a variant calling program or evidence such as dbSNP) are estimated using a latent class analysis (LCA) approach similar to the approach previously used to combine sets of gene prediction [6] and to infer orthologous genes from different genomes [7]. Briefly, this approach assumes a multinomial probability model that uses the number of observed counts for each possible combination of evidence sources that detect a given SNP to calculate the underlying parameters for each evidence source: the background frequency of true cases (alpha), and the independent and identically distributed (iid) false positive and false negative error rates of each evidence source. This LCA model is implemented using a fully Bayesian Markov Chain Monte Carlo (MCMC) simulation using the R2JAGS R package [http://cran.r-project.org/web/packages/R2jags/index.html]. For each of the three parameters to be estimated (the background frequency of true cases, and the false positive or false negative rates), a random value is selected from a beta distribution with shape parameters a of 1 and b of 2 for 120,000 iterations to yield an estimated value for each of these three parameters.

**Figure 1:**
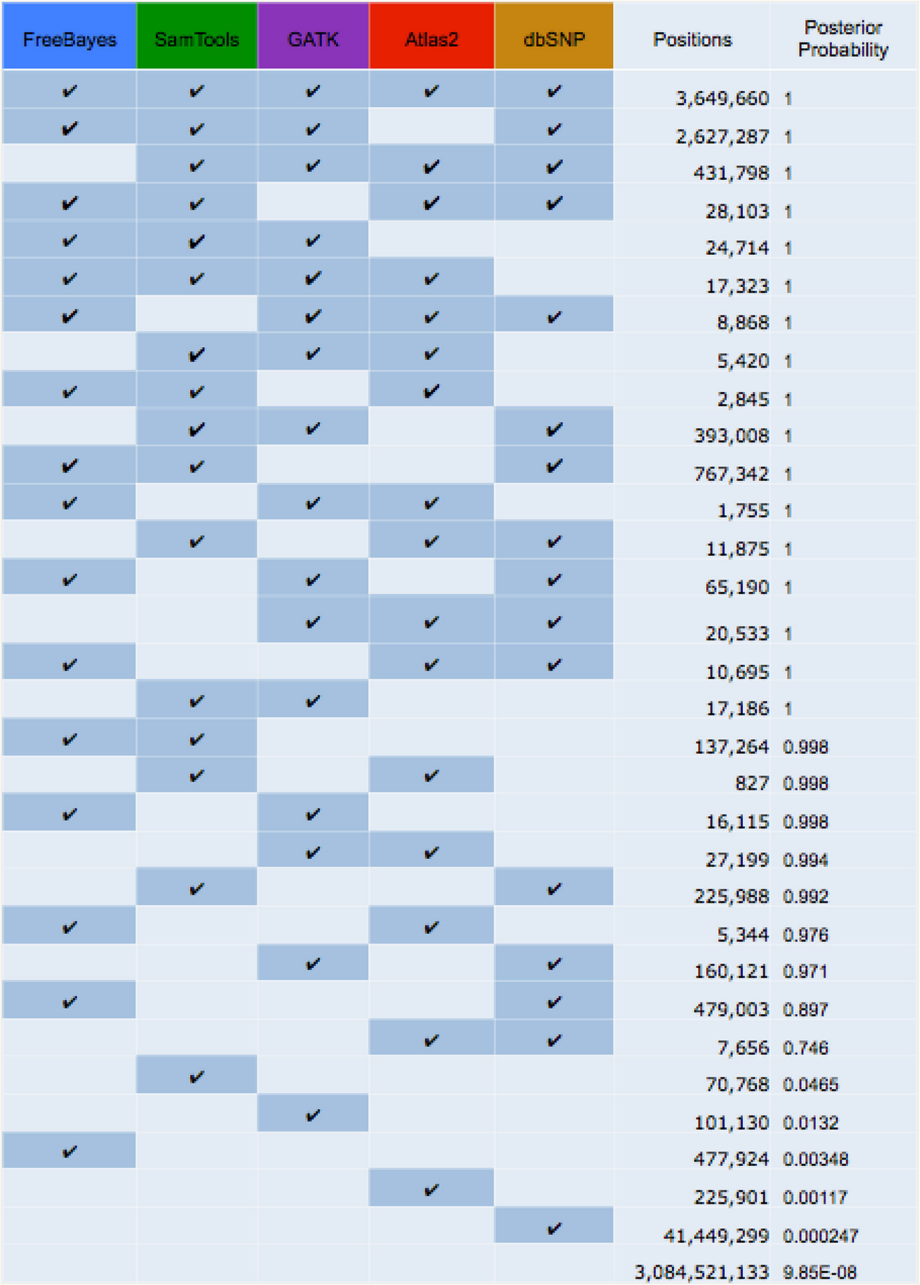
**Contingency table and posterior probabilities for SNP variant detection programs.** Variants were detected jointly on ten samples from The 1000 Genomes Project using FreeBayes, SamTools, GATK, and Atlas as described in Methods. For each possible combination of agreement amongst the variant calling programs and dbSNP, the observed number of SNP variant positions and the posterior probability calculated by BAYSIC is shown.

The posterior probability for each possible combination of agreement amongst the evidence sources (as in Figure 1) are then calculated as:

$$\frac{\theta \prod_{i=1}^{r}\beta_{i}^{1-x_{i}}{\left(1-\beta_{i}\right)}^{x_{i}}}{\theta \prod_{i=1}^{r}\beta_{i}^{1-x_{i}}{\left(1-\beta_{i}\right)}^{x_{i}}+\left(1-\theta \right)\prod_{i=1}^{r}\alpha_{i}^{x_{i}}{\left(1-\alpha_{i}\right)}^{1-x_{i}}}$$

where r is the number of evidence sources used, α_i_ is the false positive rate for the i^th^ program, β_i_ is the false negative rate for the i^th^ evidence source, and θ is the estimate of rate of overall SNP occurrence, x_i_ is 0 or 1 depending on whether the i^th^ evidence source called a SNP at the given location. For each variant, a posterior probability is determined based on which evidence source(s) detected the variant, and the posterior probability cutoff is applied to yield a set of integrated variant calls.

BAYSIC is implemented as a Perl script that receives input parameters from the user (VCF files, posterior probability cutoffs, and output file names). The Perl script invokes a separate R script, which computes the α, β and θ parameters and the posterior probabilities for each possible combination of programs. The Perl script then determines the posterior probability for each SNP variant based on which callers detected the variant, and writes out to a VCF those variants whose posterior probability is greater than the posterior probability cutoff specified by the user or a default value of 0.8 if no cutoff was specified.

## Methods

### Detection of genome variants using samples from the 1000 genome project

To detect genome variants, GATK version 2.1-9 [8], Atlas version v1.4.3 [9], Samtools version 0.1.18 (http://samtools.sourceforge.net/) and FreeBayes version 0.9.7 [10] were used. BAM files for the following ten samples were downloaded and used as input for the four variant calling programs above: NA12341, NA18566, NA12489, NA18959, NA18498, NA19007, NA18519, NA19700, NA18532 and NA19819. VCF files output by these programs as well as a VCF for dbSNP build version 137 were used as input for BAYSIC.

### Measurement of sensitivity and specificity using data from the 1000 genome project

Sensitivity of each variant detection program was measured as the percent of SNPs detected by the given program that were confirmed by orthogonal technology (OmniChip) detected by each program. Specificity for each program was measured as the ratio of transitions to transversion (Ti/Tv) for the set of SNP variants produced by each program using VCFTools [11].

### Detection of clinically associated genome variants in a previously verified sample

Peripheral blood was taken from a male patient diagnosed with vanishing white matter leukodystrophy, as well as from the unaffected father, mother and sister. Genomic DNA was extracted from each sample using standard protocols, and exome capture was carried out using Illumina’s TruSeq technology according to the manufacturer’s protocols. Enriched exome libraries were then subjected to next generation sequencing using standard TruSeq sample preparation protocols from the manufacturer (Illumina), and paired end sequencing was carried out on an Illumina HiSeq. Image analysis and base calling was carried out using CASAVA 8.2. BWA was used to align sequence reads to reference genome hg19 with subsequent processing by Samtools (http://samtools.sourceforge.net) and Picard (http://picard.sourceforge.net/) to ensure proper file formatting. Alignments were then recalibrated and realigned using GATK [8].

### Detection of somatic mutations and measurement of sensitivity and specificity in tumor versus normal pair data

Using sequencing data from tumor and normal pair from a single patient available in COSMIC (patient PD3404), we produced somatic mutation calls using MuTect [12], VarScan2 [13], Shimmer [14] and Strelka [15]. These four sets of somatic mutation calls were combined using BAYSIC with a posterior probability cutoff of 0.8. Sensitivity was approximated as the overall number of somatic mutations detected by the program, and specificity was measured as percent of somatic mutation calls produced by the program that were present in COSMIC version v63 [16].

## Results and discussion

### Overview of BAYSIC algorithm

Several programs exist for the detection of genome variants such as SNPs and insertions and deletions (http://sourceforge.net/p/atlas2/wiki/Atlas2%20Suite/) [9,17,18]. Previous studies have demonstrated that the agreement between sets of genome variants produced by these methods is poor [19]. The impact of this disagreement among callers on the analytical validity and clinical utility of genomic sequencing is obvious.

**Figure 2:**
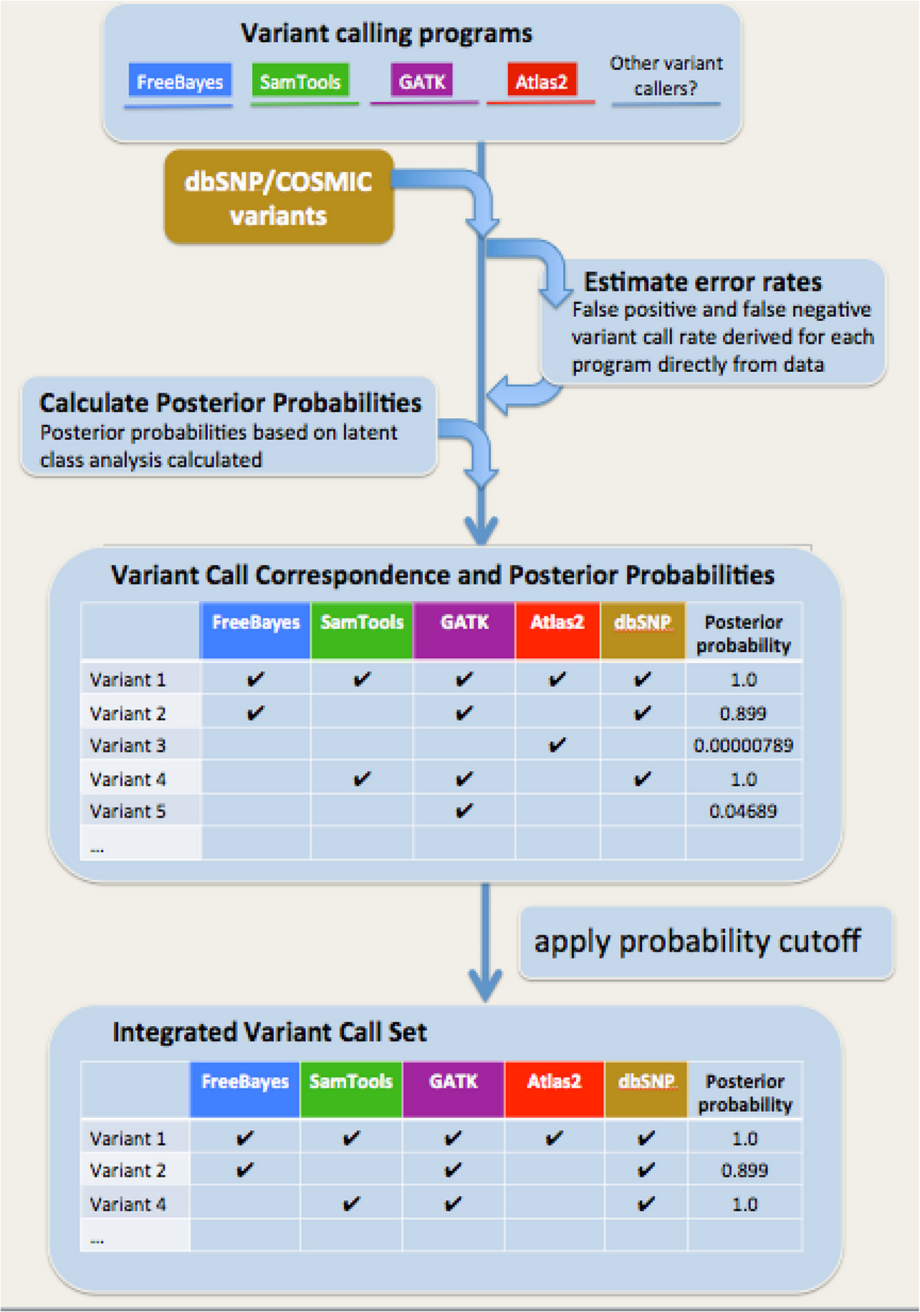
**Overview of BAYSIC algorithm.** Sets of variant calls produced from one or more programs are input in VCF format. Optionally, variants from third party databases are included as additional sources of information, e.g. dbSNP for normal variant calling and COSMIC for somatic mutation calling. False positive and false negative error rates are estimated using Markov Chain Monte Carlo simulation, and a posterior probability is calculated for each possible combination of agreement between the variant calling programs (see Methods). Finally, variants whose posterior probability is greater than the cutoff specified by the user (default value = 0.8) are output to generate a set of integrated variant calls.

BAYSIC allows users to combine two or more sets of genome variants. The user supplies one or more VCF files containing the sets to be combined and a posterior probability cutoff based on the user’s tolerance for false positive and false negative errors (Figure 2). Optionally, the user may also supply a set of known variants from third party databases in order to increase accuracy, such as dbSNP or COSMIC. The rate of false positive and false negative errors for each set of variant calls are estimated based on the input data using a MCMC simulation, and the posterior probability for each possible combination of agreement between the sets of calls is determined (see Methods). The posterior probability cutoff is then applied, and each variant that passes the cutoff is written out to a new VCF file containing the integrated set of variant calls.

### Sensitivity and specificity of BAYSIC algorithm

To evaluate BAYSIC, we first detected genome variants using ten samples from the 1000 Genomes Project [20] using GATK version 2, FreeBayes, Atlas and SamTools.

**Figure 3:**
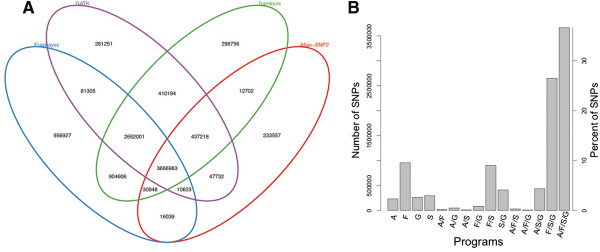
**Agreement amongst variant calling programs.** Variants were detected jointly on ten samples from The 1000 Genomes Project using FreeBayes, SamTools, GATK, and Atlas, as in Figure 1. **A**. For each SNP, agreement amongst the variant calling programs was calculated. The number of SNPs detected by each of the programs is indicated by the number in the enclosing ellipses. **B**. Agreement amongst the variant calling programs displayed as a barplot. A = Atlas, F = FreeBayes, G = GATK, S = Samtools. Left-hand y-axis indicates number of SNPs detected by the programs denoted on the x-axis. Right-hand y-axis indicates the percent of all SNPs detected by the programs denoted on the x-axis.

As seen previously [19], there was alarming disagreement among the four sets of genome variant calls. Many SNPs were present only in one set (296,756; 956,927; 233,557; 261,251 for SNP detected only by SamTools, FreeBayes, Atlas and GATK, respectively) (Figure 3A). Further, only 36.8% (3,666,983) of calls were present in all four sets (Figure 3B), and only 82.5% (8,222,619) of SNPs were present in two or more sets.

We next combined these four sets of variant calls using BAYSIC. We used as input to BAYSIC the VCF files generated from GATK, FreeBayes, Atlas and Samtools as well as a VCF containing variants from dbSNP version 137. The number of positions and posterior probabilities for each possible combination of variant callers and dbSNP are shown in Figure 1. For this particular dataset, SNPs detected by any two prediction methods (including dbSNP) would have passed the 0.8 posterior probability threshold with the exception of a prediction by Atlas and dbSNP.

To evaluate the performance of BAYSIC in comparison to existing variant calling programs, we measured the sensitivity and specificity of each method. Sensitivity was measured as the percent of SNPs detected using an orthogonal technology – array based genotyping (OmniChip) [21]. Specificity was measured as the ratio of transitions and transversions (Ti/Tv), previously demonstrated to be approximately 3 in coding regions and approximately 2 in non-coding regions for true positive SNPs [22,23], but 0.5 for false positive SNPs [24]. Contamination of SNP call sets with many false positives results in a Ti/Tv closer to 0.5, while fewer false positives will result in a value close to the normal value of Ti/Tv: 3 or 2 for coding regions and non-coding regions, respectively. Ti/Tv may therefore be used as a measure of specificity since it is proportional to the rate of false positive SNP detection.

**Figure 4:**
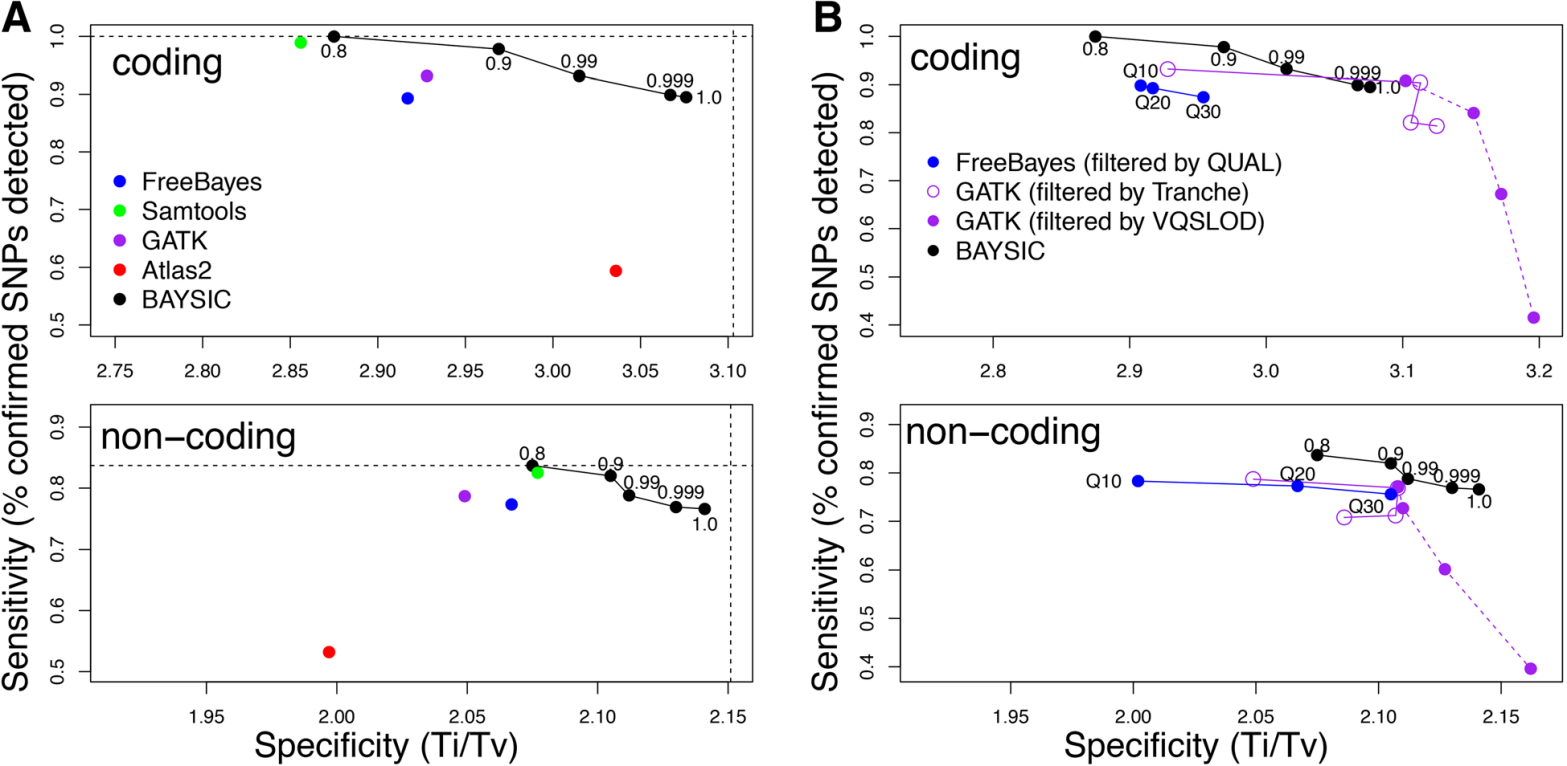
**Sensitivity and specificity of BAYSIC and other variant calling programs. A**. Improvement of sensitivity and specificity of BAYSIC compared with input variant calling programs used with default parameters. SNP variants were detected jointly on ten samples from The 1000 Genomes Project using FreeBayes, SamTools, GATK (low quality filtered) and Atlas as in Figure 1, and the four variant sets and dbSNP were combined using BAYSIC. Sensitivity of each of the variant calling programs and BAYSIC was measured as the percent of SNPs confirmed by an orthogonal platform (SNP-chip) that was detected by the given program. Specificity was measured as the transition/transversion ratio (Ti/Tv) of all SNP variants called by each program. The sensitivity and specificity for SNPs in coding (top) and non-coding regions (bottom) are shown. Numbers accompanying black symbols indicate posterior probability cutoff used for generating the BAYSIC integrated variant sets. Horizontal dashed line indicates the specificity of the intersection of the four sets of variant predictions produced by FreeBayes, SamTools, GATK and Atlas. Vertical dashed line indicates sensitivity of the union of the four sets of variant predictions produced by FreeBayes, SamTools, GATK and Atlas. **B**. BAYSIC sensitivity and specificity compared with variant calling programs with continuous estimates of variant probability. Variants were detected using FreeBayes and GATK with varying stringency by applying cutoffs based on quality scores (for FreeBayes) or either Tranche scores or VQSLOD scores (for GATK). Sensitivity and specificity are shown for FreeBayes with cutoffs of Q10, Q20 (blue points) and GATK with Tranche cutoffs (open purple points, no cutoff, Tranche90, Tranche99 and Tranche99.9) or VQSLOD cutoffs (closed purple points, VQSLOD cutoffs of 0, 2.9, 4.4 or 5.4 from left to right). Sensitivity and specificity of BAYSIC using FreeBayes, Samtools, GATK and Atlas with default parameters as input are shown for comparison.

As expected, as a more stringent posterior probability cutoff was applied, the specificity of the variant set produced by BAYSIC improved at the expense of a slight decrease in sensitivity. The sensitivity of BAYSIC (using a posterior probability cutoff of 0.8 and using as input the set of SNP calls from GATK, FreeBayes, Atlas and Samtools, along with SNPs from dbSNP) in detecting SNPs in coding regions was identical to the union of the set of all SNPs detected by these same four programs, which represents maximal sensitivity (Figure 4A, compare horizontal dashed line with black circle labeled 0.8). Similarly, the sensitivity of BAYSIC calls in noncoding regions was nearly identical to the union of all variant calls. For SNPs in coding regions, BAYSIC, using a default posterior probability cutoff of 0.8, detected 16,978 (100%) of the OMNI CHIP-confirmed SNPs in coding regions, and the union of the call sets, combining calls from each of the callers detected 16,978 (100%) of the OMNICHIP-confirmed SNPs in coding regions. For SNPs in non-coding region, BAYSIC detected 903,984 (83.7%) of OMNICHIP-confirmed SNPs, while the union of all SNPs detected by these four programs yielded 903,998 (83.7%) of these OMNI CHIP-confirmed SNPs. As the posterior probability cutoff applied to the BAYSIC set was increased from 0.8 to 1.0, the specificity increased while the sensitivity decreased (Figure 4A, black circles). At a posterior probability cutoff of 1.0, the specificity approached the specificity of the intersection of the set of SNPs called by all four programs with default parameters, which represents maximal specificity (Figure 4A, vertical dashed lines).

BAYSIC improved the sensitivity and specificity of the SNP detection programs used as input to BAYSIC. In detecting SNPs in non-coding regions, BAYSIC with the default posterior probability cutoff of 0.8 was more sensitive than FreeBayes, Samtools and Atlas2 and GATK with no filter applied, and more specific than FreeBayes, GATK and Atlas2, and comparable in specificity to Samtools (Figure 4A, bottom panel). In detecting SNPs in coding regions, BAYSIC with the default posterior probability cutoff of 0.8 was more sensitive than FreeBayes, Samtools and Atlas2 and GATK with the low quality filter applied, and higher in specificity than GATK and Samtools in non-coding regions. FreeBayes, Atlas2 and GATK with low quality filter applied, however, were higher in specificity in coding regions than BAYSIC with the default posterior probability cutoff of 0.8. When the BAYSIC posterior probability threshold was increased to 0.9, the specificity of BAYSIC in coding regions exceeded Samtools, FreeBayes and GATK with low quality filter, and the specificity of BAYSIC in non-coding regions exceeded all 4 input call sets. Samtools sensitivity was slightly higher than BAYSIC with a posterior probability cutoff of 0.9, and Atlas2 coding region specificity is slightly higher than BAYSIC with posterior probability set to 0.9.

Since other variant calling programs offer filtering options to increase the specificity of SNP detection at the expense of sensitivity similar to the posterior probability cutoff available in BAYSIC, we compared the performance of these filtering options with those of BAYSIC. BAYSIC performed favorably compared with GATK SNP call sets filtered using the Tranche and VQSLOD options, and also with FreeBayes SNP call sets filtered using the QUAL score. In SNPs occurring in non-coding regions, BAYSIC (run with input from the Samtools, FreeBayes, Atlas and GATK with default parameters) with increasing posterior probability cutoffs described a curve that was above and to the right of curves for GATK with increasingly stringent Tranche and VQSLOD filtering, and FreeBayes with increasingly stringent QUAL score filtering (Figure 4B, lower panel). In SNPs occurring in coding regions, BAYSIC (using Samtools, FreeBayes, Atlas and GATK with default settings as input) with increasingly stringent filtering described a curve that was above and to the right of FreeBayes using QUAL filtering, and more sensitive and specific than GATK using Tranche filtering when BAYSIC was run with a posterior probability p > 0.99 (Figure 4B, top panel). At p > 0.999 and p = 1.0, BAYSIC was slightly more sensitive but less specific than GATK Tranche 99 and Tranche 99.9, and less sensitive and specific than Tranche 90. Compared with GATK using with VQSLOD filtering, BAYSIC (again using as input Samtools, FeeBayes, Atlas and GATK with default parameters) was generally more sensitive, but less specific.

**Figure 5:**
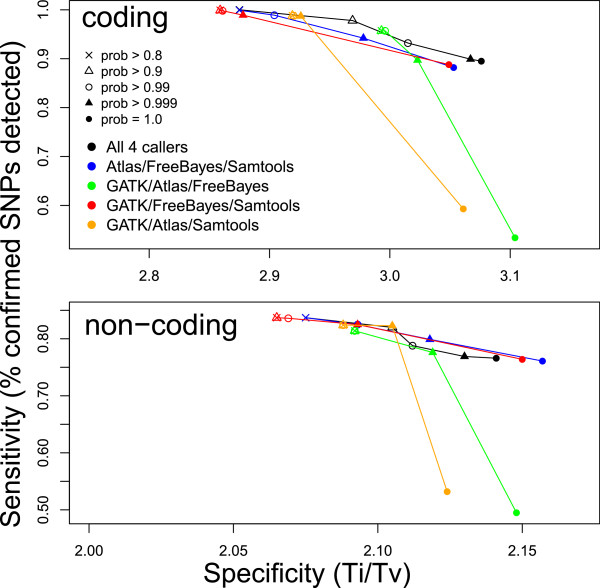
**Effect of variant calling programs used as input on sensitivity and specificity of BAYSIC.** SNP variants were detected with BAYSIC using as input all possible combinations FreeBayes, SamTools, GATK and Atlas. The sensitivity and specificity of each set was then measured as in Figure 4 for SNPs occurring in coding regions (top) or non-coding regions (bottom). The sensitivity and specificity for each combination of input call sets and for a range of posterior probability cutoffs is shown.

To assess the importance of each individual variant calling program used as input to BAYSIC, we next investigated the effect of leaving out one of the four variant calling programs (GATK, Atlas, FreeBayes and Samtools) on the sensitivity and specificity of BAYSIC. Overall, the sensitivity and specificity of BAYSIC using as input any three of variant caller programs were comparable to the specificity using all four variant calling programs (Figure 5, top and bottom, compare colored curves to black curves), although the sensitivity of two sets (GATK/Atlas/FreeBayes and GATK/Atlas/Samtools) dropped markedly when applying the highest posterior probability cutoff of 1.0. Also, applying the same posterior probability cutoff to sets of variant produced by different programs resulted in different sensitivity and specificity (for example, compare filled triangles). It is also noteworthy that the inclusion of the GATK set as input into BAYSIC had little effect on the sensitivity and specificity of the resulting integrated SNP set produced by BAYSIC. The sensitivity and specificity of BAYSIC using Atlas, FreeBayes and SamTools as input was comparable to that of BAYSIC using Atlas, FreeBayes, SamTools and GATK as input (Figure 5, compare black and blue curves).

### BAYSIC analysis of exome data from a subject with a previously detected known clinically relevant mutation

To evaluate the performance of BAYSIC in a clinical setting, we tested the ability of these programs to detect a compound heterozygous mutation (rs113994057 and rs113994049) in the EIF2B5 gene, mutations in which have been shown to be causative for vanishing white matter leukodystrophy [25]. The genotypes at these two SNPs were characterized in a family consisting of one child affected with vanishing white matter leukodystrophy, two healthy parents and one healthy child using standard clinical genetic testing by Sanger sequencing. (In this family, Sanger sequencing indicates that two of the total of eight alleles for all individuals for rs113994057 are ALT, and three of the eight alleles for rs113994049 are ALT). Whole exome sequence data was obtained for these same four individuals, and sequence variants were detected using GATK, Atlas, Samtools, FaSD (http://wanglab.hku.hk/FaSD/) and SNP calls from these four programs were then integrated using BAYSIC. BAYSIC with a default posterior probability cutoff (0.8) correctly identified the genotypes of both SNPs in all family members. Both SNPs were identified by three of the four callers, and the posterior probability calculated by BAYSIC for each SNP was greater than 0.999, far above the default cutoff of 0.8. GATK and Samtools were able to detect the risk allele in all four family members, but GATK did so only when operating with the most relaxed filtering option (LQF). Atlas2 only detected one (rs113994057) of the two causal SNPs (Table 1). FaSD did not predict genotypes for healthy family members; failing to detect the variants as candidates. Therefore, results from Samtools or GATK with low quality filter would have lead researchers to correctly identify the compound heterozygous SNPs. However, if researchers were to use any of the more stringent filtering options commonly used in GATK, e.g., Tranche 90, 99, 99.9 or a PASS filter, these SNPs would not have been detected. Because one of the two SNPs is rare, our integrated approach provides additional confidence for researchers interested in detecting rare SNPs.

**Table 1:** Identification of independently verified, disease causative SNPs

**SNP**	**Atlas**	**GATK**	**Samtools**	**FaSD**	**BAYSIC**
rs113994057	Detected	Detected (Tranche90)	Detected	Not detected	Detected
rs113994049	Not detected	Detected (LQF)	Detected	Detected	Detected

SNP variants were detected in samples from four individuals from a single family with one individual affected with white matter leukodytrophy 2 healthy parents and a healthy sibling. For each of two causative SNPs in the EIF2B5 gene associated with white matter leukodystrophy (rs113994057 and rs113994049), the ability of Atlas, GATK, Samtools, FaSD or BAYSIC (with the default posterior probability cutoff of 0.8) to detect each variant is shown. For GATK, the most stringent filter that could be applied and still detect each variant is shown in parentheses. “Detected” indicates that the SNP was predicted for one of the four subjects.

### Using BAYSIC to combine sets of somatic mutation calls produced with tumor/normal pair data

A common application of genome sequencing is to sequence samples taken from normal and tumorous tissue and detect somatic mutations that may be involved in cancer [26]. Many programs exist to detect somatic mutations, but as with programs for detecting SNP variants, the agreement of these programs is poor [27]. The problem of combining these sets of somatic mutations is analogous to the problem of combining disparate sets of SNPs produced by different SNP detection programs.

We applied BAYSIC to this related problem of combining disparate sets of somatic mutation calls. Using sequencing data from tumor and normal pair from a single patient available in a catalog of previously observed somatic mutations (COSMIC; patient PD3404), we produced somatic mutation calls using MuTect [12], VarScan2 [13], Shimmer [14] and Strelka [15], and then combined these four sets of somatic mutation calls using BAYSIC with a default posterior probability cutoff of 0.8.

**Figure 6:**
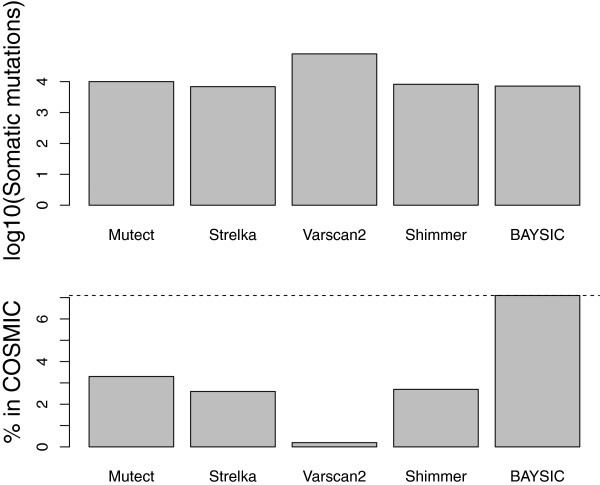
**Combining somatic mutation calls from tumor/normal pair samples using BAYSIC.** Somatic mutations from exome data from a single patient were predicted using Mutect, Strelka, Varscan2 and Shimmer and these four sets of somatic mutation calls were combined using BAYSIC with a posterior probability cutoff of 0.8. As a measure of sensitivity, the number of somatic mutations predicted by each caller that are present in COSMIC, a database of somatic mutation calls from other samples, is shown (top). As a measure of specificity, the percent of each set of somatic mutation calls that are present in COSMIC is shown (bottom). A horizontal dashed line indicating the percent of BAYSIC somatic mutational calls present in COSMIC is shown.

As a measure of specificity, we determined the overall number of somatic mutations detected by each program that were present in COSMIC (a database of previously observed somatic mutations). MuTect, VarScan2, Shimmer, Strelka and BAYSIC, using as input the sets of somatic mutation produced by all four callers, detected 330, 165, 222, 165 and 510 somatic mutations that were present in COSMIC, respectively (Figure 6, Table 2); this translates to 3.3%, 0.2%, 2.7%, 2.6% and 7.1% of the total SNPs. Using this as a measure, BAYSIC therefore improves the specificity of all four callers used as input. BAYSIC predicted a lower number of somatic mutations (7,914) compared to MuTect, VarScan and Shimmer (9,977; 79,313; 8,222, respectively), but more than Strelka (6,887). If the overall number of somatic mutations is taken as a measure of sensitivity, the sensitivity of BAYSIC is lower than Mutect and Shimmer, much lower than VarScan and higher than Strelka. In general, each of the four individual callers is highly concordant with the genotype predicted by the genotype chip, one measure of accuracy. For all programs, all somatic mutations that occurred at positions present on the genotype chip predicted genotypes in agreement with the genotype chip (Table 2).

**Table 2:** Comparison of somatic mutation prediction methods

	**MuTect**	**Strelka**	**VarScan2**	**Shimmer**	**BAYSIC**
# of somatic mutations	9977	6887	79313	8222	7194
Positions on chip	2	2	120	23	3
Agreement with chip (SM on chip/SM with genotype agreeing with chip)	100% (2/2)	100% (2/2)	100% (104/104)	100% (10/10)	100% (3/3)
# in COSMIC	330	179	165	222	510
% in COSMIC	3.3%	2.6%	0.2%	2.7%	7.1%
% unique somatic mutations	36%	8%	83%	51%	-
# of somatic mutations causing coding changes	58	58	458	113	164

Somatic mutations from exome data from a single patient were predicted using Mutect, Strelka, Varscan2 and Shimmer and these four sets of somatic mutation calls were combined using BAYSIC with a posterior probability cutoff of 0.8. The total number of somatic mutations detected by each program, as well as the agreement of these somatic mutations with those COSMIC and with sets determined by chip are shown.

## Conclusions

Clinical applications of genomics demand reliable detection of real variants and discrimination and rejection of false alarms due to sequencing error, low sequence coverage or low allelic variant fraction. Accordingly, the utility of genomic medicine will be improved by better methods for accurately identifying SNPs and other genomic variants.

Our analyses support our initial hypothesis: BAYSIC variant calls demonstrated improved variant detection accuracy and superior receiver operating characteristics compared to the variant call methods used as input for BAYSIC.

Importantly, BAYSIC will accept as input any number of alternative variant detection algorithms, allowing the user to combine methods that emphasize sensitivity with methods that enhance specificity and achieve overall gains in detection accuracy. As the sensitivity *or* specificity of input call sets improve, the sensitivity *and* specificity of BAYSIC variant calls also increases.

Likewise, BAYSIC may be used to focus on specific types of variant detection problems such as somatic mutations in cancer, and achieves similar gains in receiver operating characteristics compared to the individual somatic variant calling algorithms used as input. Another program was recently described to combine somatic mutation calls [28]. Future work will determine the relative performance of BAYSIC compared with this program, and assess how inclusion of improved somatic mutation call sets, as input to BAYSIC, affects BAYSIC’s overall performance in somatic mutation detection.

It is possible that the degree of improvement offered by BAYSIC in combining sets of germline SNP variant calls compared to somatic mutation calls is explainable by the different error rates in these two different experimental settings. That is, germline SNP discovery has very low false positive and low false negative rates relative to somatic mutation calls, with generally good sensitivity and specificity [12,28]. Therefore, producing a consensus set of germline SNP variants with BAYSIC provides marginal but noticeable improvements to both sensitivity and specificity (Figures 4 and 5). In contrast, somatic mutation discovery has very high false positive (and possibly also high false negative) rates, with poor specificity (and perhaps also poor sensitivity). Producing a consensus SNP set using BAYSIC therefore makes dramatic improvements to specificity without losing sensitivity (Figure 6).

It is possible that correlations between the errors in the sets of variant calls used as input to BAYSIC could result in false positive errors in the integrated variant set produced by BAYSIC. To address this, future versions of BAYSIC will measure the bivariate residuals after latent class analysis is performed, and will penalize the significance of input sets that are highly correlated [7].

BAYSIC currently only integrates sets of SNP variant calls. Future work will expand this to include other sorts of variants such as insertions/deletions (indels), and additional modifications to facilitate improved performance in somatic mutation detection.

## Availability and requirements

**Project name:** BAYSIC

**Project home page:**http://genformatic.com/baysic

**Operating systems:** Linux, OS X, Windows

**Programming languages:** Perl, R

**Other requirements:** JAGS, JSON File::Temp Getopt::Long List::Util File::Next Test::Warn File::Slurp PerlIO::gzip File::Which local::lib

**License:** Free for academic use, license needed for commercial use

## Competing interests

BC, DW and JR own an interest in Genformatic, LLC, a company which performs sequencing and sequence data analysis as a service. This does not alter the authors’ adherence to the journal’s policies on sharing data and materials.

## Author’s contributions

JR, AM, BC, DW, NM and JZ conceived, designed and executed experiments. NM and JZ provided access to clinical data. AM and JR designed the algorithm and wrote software. All authors contributed to preparation of the manuscript.
